# Microeconomic Surplus in Health Care: Applied Economic Theory in Health Care in Four European Countries

**DOI:** 10.3389/fphar.2013.00017

**Published:** 2013-02-18

**Authors:** S. Walzer, M. Nuijten, C. Wiesner, K. Kaier, P-O. Johansson, S. Oertel

**Affiliations:** ^1^MArS Market Access and Pricing Strategy UG (h.b.)Weil am Rhein, Germany; ^2^Ars Accessus MedicaAmsterdam, Netherlands; ^3^F. Hoffmann-La Roche AGBasel, Switzerland; ^4^Institute of Medical Biometry and Medical Informatics, University Medical Center FreiburgFreiburg, Germany; ^5^Stockholm School of EconomicsStockholm, Sweden

**Keywords:** microeconomic surplus, convenience, health care, health care policy

## Abstract

**Introduction:** In economic theory economic surplus refers to two related quantities: Consumer and producer surplus. Applying this theory to health care “convenience” could be one way how consumer benefits might manifest itself.

**Methods:** Various areas of economic surplus were identified and subsequently screened and analyzed in Germany, Spain, The Netherlands, and the UK: Cesarean births, emergency room visits (nights or weekends), drug availability after test results, and response surplus. A targeted literature search was being conducted to identify the associated costs. Finally the economic surplus (convenience value) was calculated.

**Results:** The economic surplus for different health care areas was being calculated. The highest economic surplus was obtained for the example of response surplus IVF-treatments in The Netherlands.

**Conclusion:** The analyzed examples in this article support the underlying hypothesis for this research: “Value of convenience defined as the consumer surplus in health care can be shown in different health care settings.” Again, this hypothesis should be accepted as a starting point in this research area and hence further primary research is strongly recommended in order to fully proof this concept.

## Health Care Market: An Introduction

In recent years, the introduction of new innovative medicinal products has become increasingly difficult as a result of the budget pressure, the introduction of more complicated procedures and higher demands on added value of medicinal products and other therapies. For the most part, those measures have relied on budgeting or price controls, including negotiated prospective budgets for hospitals, centralized negotiated budgets for ambulatory physicians including drug prescriptions, and limitations on payments for particular medications. Autonomous behavior by prescribers is restricted to follow national clinical guidelines, local formularies and/or local agreements between prescribers and health insurers, who sanction on deviant prescription behavior or reward proper prescription behavior. Though every country in Europe has its own specific cost containment measures and hurdles for market access, the changes have a similar impact on each new medicinal product introduction in Europe: more and more refusal or restrictive access to new therapies following negative reimbursement decisions. Because those traditional central cost containment measures were only partially successful, due to lack of incentives, the health authorities in Europe started to establish incentives for efficient health care delivery. Although there is large variety between the various countries, there are two related trends: the implementation of market mechanisms and decentralization of the health care decision-making process (Nuijten et al., 2011). The aim of those reforms is to control increasing health care costs, which has become an important part of the collective economic burden.

### Concept of convenience

In Webster’s Dictionary (Report of the Consumer Healthcare Products Association’s Clinical/Medical Committee, 2010), convenience is defined as “anything that adds to one’s comfort or saves work; useful, handy, or helpful device, article, service, etc.” Various conceptual frameworks for convenience have been developed (Report of the Consumer Healthcare Products Association’s Clinical/Medical Committee, 2010). In general, all of these frameworks address the time and energy or effort consumers spend deciding on, accessing, transacting for, and benefiting from a product or service.

Convenience in health care could also be linked to a strong microeconomic basis, the theory of economic surplus (Zweifel et al., 2009). In economic theory economic surplus refers to two related quantities:

○ Consumer surplus which is the monetary gain obtained by consumers because they are able to purchase a product for a price that is less than the highest price they would be willing to pay.
○ Producer surplus which is the amount that producers benefit by selling at a market price that is higher than the least they would be willing to sell for.

On a standard supply and demand diagram, consumer surplus is the area above the equilibrium price of the good and below the demand curve. This reflects the fact that consumers would have been willing to buy a single unit of the good at a price higher than the equilibrium price, a second unit at a price below that but still above the equilibrium price, etc., yet they in fact pay just the equilibrium price for each unit they buy.

Likewise, in the supply-demand diagram, producer surplus is the area below the equilibrium price but above the supply curve. This reflects the fact that producers would have been willing to supply the first unit at a price lower than the equilibrium price, the second unit at a price above that but still below the equilibrium price, etc., yet they in fact receive the equilibrium price for all the units they sell.

Even though that health care markets are normally being defined as imperfect markets due to different specifications of it (e.g., moral hazard problem) the theory of economic surplus can still be applied (Cabral, 2002; Zweifel et al., 2009). In standard microeconomic text books it is well described that consumer (and producer) surpluses can be shown in any market. Just the value a consumer or producer can skim might be different due to the underlying market mechanisms.

Applying the theory to health care, “convenience” could be one way how consumer benefits might manifest itself. We have analyzed the existence of microeconomic surpluses in different health care areas in Germany, Spain, The Netherlands, and the UK.

## Materials and Methods

The underlying hypothesis for this research was as follows: “Value of convenience defined as the consumer surplus in health care can be shown in different health care settings.” As this hypothesis is a starting point in this research area the analysis have been solely based on published literature and otherwise publicly available information. Further primary research might be needed in order to proof the concept.

In order to get an overview of existing convenience areas in healthcare a targeted literature search in standard databases for medical and economic literature was being conducted: PubMed, Medline, EconLibrary, and EconLit. Furthermore a general search in the internet using Google was applied. Keywords being used were: microeconomics, convenience, health care, economic surplus, microeconomic surplus, patient surplus. As a result there was basically no manuscript available analyzing convenience in healthcare as an example of microeconomic surplus. Subsequently we have conducted a review of healthcare systems using the following approach in order to identify different areas of potential convenience in the systems. Different exemplary treatments, processes, or services a healthcare system delivers to patients were defined.

- Cesarean births: A reason for discussion of this area is the increased incidence of cesarean births in the developed markets without an increased medical need (Robson et al., 2009).
- Emergency room visits during nights and weekends are also a potential area of convenience in the system as the cost are generally higher for the service provider (hospital) but the payment for the service is the same as during “normal” working hours.
- Drug availability after test results: Personalized healthcare is one of the key areas for all healthcare stakeholders and hence we have also discussed the potential availability of convenience for indications where a drug treatment is just available after a positive test result.
- Response surplus with the example of In vitro fertilization (IVF)-treatment: For some treatments payers restrict their payment to a maximum number of treatment cycles. In the case of infertility IVF-treatments are normally fully reimbursed (medication, procedures, and consultations) for 2–3 cycles depending on the insurance and health care system.

After the identification of the areas to be analyzed a targeted payment and cost search was conducted in order to link the different medical examples with costs and/or reimbursement tariffs dependent on the purpose of the chosen example. In order to get a European overview of convenience areas, different examples were taken from different countries: Cesarean births were analyzed in Germany, emergency room visits in the UK, drug availability after test results in Spain, and the response surplus example in The Netherlands.

## Results

### Cesarean births

Robson et al. (2009) report that the rate of cesarean section in Australia now exceeds 30% and evidence from population studies indicates that maternal requests for elective cesarean delivery might make an important contribution. In an anonymous survey they have analyzed 1,239 specialist obstetricians and 317 obstetric specialty trainees in Australia and came to the conclusions that “at least 17% of all elective cesarean sections, and slightly more than 3% of all births” were being done on request by the patient without a clear medical indication. This evidence is probably also seen in other developed markets.

We have used the German market to analyze the potential economic surplus. For the hypothetical analyses below it is especially assumed that a woman could also change her preference from a vaginal birth to a cesarean during the actual process of the birth without a medical need. The difference of a Diagnosis Related Groups (DRG) for a vaginal birth and a Cesarean birth in Germany was calculated (see Table 1). The base rate fee of €2’800 was multiplied by the DRG value which resulted in the actual DRG value for the two analyzed DRGs. This difference (€828 per case) represents the average financial burden per single non-medical driven Cesarean birth covered by the insurer. According to the theory the microeconomic surplus for the respective patient, however, is represented by the difference of the potential willingness to pay for a non-medical driven Cesarean birth and the co-payment. Willingness to pay was not elicited through experiments which would obviously be a better (although still imperfect) way to quantify the consumer surplus, which the patients are collectively gathering as a result of the too low market price of their healthcare consumption. In the absence of this value for willingness to pay, we quantified the producer surplus in a simplistic way, as we were looking at the incremental reimbursement (revenue) for the provider, without looking at the collective incremental costs for Cesarean vs. vaginal delivery. It is probably fair to assume that the (individual) producer surplus per provider will not amount to the entire €828 per case.

**Table 1:** **DRGs in Germany for vaginal and cesarean births without complications**.

DRG	Description	Relation to base rate fee	Mean stay in hospital (days)
O01H	Primary section cesarean without complications, pregnancy duration of more than 33 weeks, without complex diagnosis	0.803	4.8
O60D	Vaginal birth without complications	0.507	3.3

### Emergency room visits

Emergency room visits during nights and weekends have been identified as a potential area of convenience in the healthcare system as the actual cost for the service provider (hospital) are generally higher due to higher salaries but the payment (and hence reimbursement or DRG) for the service is the same as during “normal” working hours. For the analysis of potential surpluses in the system nurses working in the emergency rooms in the United Kingdom were chosen. Nurse salaries are published by the National Health Service (NHS; UK Salary and Taxation, 2012). For this hypothetical analysis it was assumed that experienced nurses will work in emergency rooms and hence a nurse grade E (experienced nurse staff) was chosen for the analysis. As less experienced nurses have a smaller salary, the consumer surplus calculated below would then also be reduced. The annual salary of a grade E nurse can vary from £17′660 to £21′325 whereas a median salary of £19′492 was assumed. Furthermore it was assumed that all other costs to deliver an emergency room service were independent of the time of delivery of the service (day or night shift). In order to have all analysis in this paper in € an exchange rate of 0.8 was assumed (€1 = £0.8). According to the NHS nurses receive a salary premium of 30% for night times and 60% for weekends (D’Addario et al., 2010). A standard Healthcare Resource Group (HRG) value for an emergency visit was applied and the theoretical surplus was calculated applying a proxy-add-on taken from the proportionally higher wages during premium times and was ranging between €30 and €60 per case per day for nights shifts and weekends respectively. A summary table with the key assumptions is provided in Table 2. The microeconomic surplus for the patients relies on the fact that emergency room services may be seen as reserve capacities. Such reserve capacities are characterized by non-rivalry and non-excludability making them so-called public goods, whose supply usually relies on a specific public intervention.

**Table 2:** **Summary of key assumptions in the example of emergency room visits in the UK**.

Convenience area	Country	Assumption/calculation definition	Calculation	Results: Microeconomic surplus
Emergency room visits	UK	Annual salary of nurse grade E (specialist nurse): £19′492 Exchange rate: €1 = £0.81 Salary premiums for nights: 30% Salary premiums for weekends: 60% Standard HRG (emergency visit): £75	Cost coverage from daily salary for one emergency visit was calculated and compared between a “normal,” night and weekend shift	€30 surplus (night shifts) €60 surplus (weekends)

### Drug availability after test results

Non-small cell lung cancer (NSCLC) accounts for ∼85–90% of all lung cancers (D’Addario et al., 2010). It is biologically aggressive and the leading cause of cancer death in men and women. A biological and genetical variation of lung cancer is NSCLC which bears activating mutations in the tyrosine kinase domain of the epidermal growth factor receptor (EGFR). Exon 19 deletions and the L858R mutation constitute ∼90% of the EGFR mutations identified to date. EGFR MuT+ lead to structural changes, which stabilize the active form of the tyrosine kinase domain and result in a high affinity for binding EGFR tyrosine kinase inhibitors (TKIs; Carey et al., 2006). In patients with tumors that are positive for these mutations, the current data supports sensitivity to gefitinib or erlotinib (NCCN Practice Guidelines in Oncology, 2012a,b). As both therapies are approved by the European Medicines Agency (EMA) since a couple of years [European Medicine Agency (EMA) (2012a,b)] and most health care systems also approved its pricing and reimbursement one could assume that also the EGFR m+ test would be reimbursed by those payer authorities. However as an example in Spain those tests are not officially reimbursed. Until recently Astra Zeneca has paid for the test which cost around €350 per patient. This amount could also be assumed to be the surplus for the Spanish NHS as neither of the two drug therapies will be reimbursed if no positive test result is available. This could be a quite high amount as both therapies cost around €2′000 per pack (Consejo General de Colegios Oficiales de Farmacéuticos, 2012). The microeconomic surplus is more difficult to determine: From the perspective of Astra Zeneca, for example, the drug gefitinib is still under patent protection making monopoly pricing possible. Accordingly, paying for the tests may be seen as an investment that should be overwhelmed by monopoly rents due to additional gefitinib sales. The economic surplus of patients would again be represented by the difference between their willingness to pay for the additional medication (including tests) and their co-payments. Unfortunately the actual willingness to pay per patient is not known. The costs of the additional gefitinib sales, however, need to be covered by the insurer. In total those costs and hence the microeconomic surplus could be as high as €24′000 per patient assuming a treatment duration of 12 months for such patients.

### Response surplus for IVF-treatment

*In vitro* fertilization is a process by which an egg is fertilized by sperm outside the body: *in vitro*. IVF is a major treatment for infertility when other methods of assisted reproductive technology have failed (National Collaborating Centre for Women’s and Children’s Health, 2004). IVF success rates are the percentage of all IVF procedures which result in a favorable outcome. Depending on the type of calculation used, this outcome may represent the number of confirmed pregnancies, called the pregnancy rate or number of live births, called the live birth rate. Due to advancement in reproductive technology, the IVF success rates are substantially better today than they were just a few years ago. The most current data available in the United States is a 2009 summary complied by the Society for Reproductive Medicine (SART) which reports the average national IVF success rates per age group using non-donor eggs (see Table 3 below; Assisted Reproductive Technology, 2009).

**Table 3:** **Pregnancy and live birth rates according to different age groups for IVF-treatment (Assisted Reproductive Technology, 2009)**.

	<35 year	35–37 years	38–40 years	41–42 years
Pregnancy rate	47.6	38.9	30.1	20.5
Live birth rate	41.4	31.7	22.3	12.6

The live birth rates using donor eggs are also given by the SART and include all age groups using either fresh or thawed eggs. The live birth rates with fresh donor egg embryos is 55.1 and with thawed donor egg embryos it is 33.8 (Assisted Reproductive Technology, 2009).

Normally the success rates for IVF-treatment in women where an initial treatment failed is dramatically lower than in other women. For simplicity reasons this is not covered within this analysis.

In most countries the reimbursement of IVF-treatment is limited to a maximum number of treatments. For example in The Netherlands three IVF–treatments are fully reimbursed (medication, procedures, and consultations) per ongoing pregnancy and another three after pregnancy (Fiddelers et al., 2009). When an initial IVF-treatment has failed, patients may pay for this initial phase by themselves in order to save one full IVF-treatment.

For the analysis it was assumed that 21.4% of IVF treated patients will be successful and hence lead to a pregnancy. Again, the microeconomic surplus for the respective patient is represented by the difference between the willingness to pay for IVF-treatments and their co-payment. As no other willingness-to-pay data are available the following results of a US-survey are taken into account for illustrative purposes: According to a US-survey among 150 potential childbearers, for example, the average willingness to pay was $17,730 (in 1992 dollars) for a 10% chance at having a child through IVF in the event of infertility (Neumann and Johannesson, 1994). In contrast, the list price for each IVF-treatment in the Netherlands is €2′048. So a maximum of €4′096 could be saved if physicians after one first successful attempt in a woman use the two remaining attempts for another woman, who failed three times.

The above mentioned examples support the underlying hypothesis for this research (“Value of convenience defined as the consumer surplus in health care can be shown in different health care settings”). Again, this hypothesis should be accepted as a starting point in this research area and hence further primary research is strongly recommended in order to fully proof this concept.

## Discussion

In health care we face very acute information problems, which make rational purchasing decisions difficult (Zweifel et al., 2009). For most products outside of health care the buyer of a product is also the one who receives the benefits of a product. This is clearly different to the situation in health care where in most countries those who pay are not the receivers of health care (patients). In addition, patients are faced with imperfect information regarding the quality of the health service they consume. In contrast to other services, there is a lack of possibility of the random sample. Whenever externalities occur, we may have market failure if not remedied by taxes or subsidies or some “Coasian” solution to the problem (Zweifel et al., 2009).

Another issue which might have had affected our findings is asymmetry of information (Zweifel et al., 2009). The consumer, the patient, has an unclear knowledge about their current disease stage and well-being. Just take the example of cesarean births: The patient has unclear knowledge about her medical well-being, but probably some preferences regarding vaginal- and cesarean births. The treating physician, however, has some better knowledge about the status due to his academic background and experience, whereas the institution paying for the cesarean birth only gets the claims and pays. The asymmetry of information between physician and patient has become much smaller, because the patient has become more knowledgeable than in the past by means of better education and media. The Internet offers (e.g., on-line patient communities) opportunities to further reduce this knowledge gap. However the increasing complexity of medical diagnosis and procedures available make obtaining accurate knowledge difficult. In addition genetic engineering technology may further increase the information gap both for patients as well as physicians vs. providers of those products or related services.

**Figure 1:**
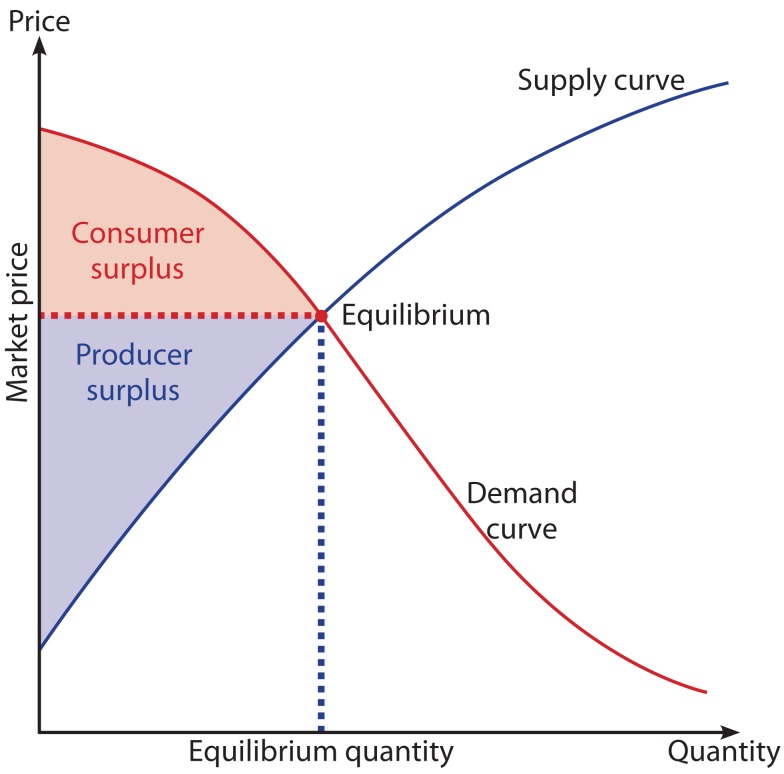
**Consumer and producer surplus in a supply-demand diagram**.

In a pure market economy supply and demand are determined by individual firms and consumers and it is the price of the commodity, which brings demand and supply into balance or equilibrium (Figure 1). The economic model of consumer behavior assumes that the consumer attempts to use his/her income in order to obtain maximum well-being or utility (consumer as maximizer) by purchasing a basket of goods and services subject to available income. It assumes that consumers know how best to increase their own welfare and therefore which goods to choose. Information is readily available on their characteristics and these can be related to the individual’s preferences. In health care markets, patients do not pay directly for treatment which might be accompanied with the problem of over-consumption due to moral hazard. Consequently the price of a health care service will not bring demand and supply into balance: The demand by the patient will not be limited by the price, and providers might have financial incentives to increase the volume of health care services. The third party, the health insurer, who is responsible for direct payment, may have some control over price, but to a much less extent on volume. A health insurance company can pass on the cost of this excess expenditure through increased contributions. The problem of patient moral hazard is compounded where providers are also given incentives to overtreat patients, especially if high reimbursements are expected. In this case neither the patient nor the provider has an incentive to contain costs. To counter this problem the literature suggests two alternative strategies: (1) the insurer may impose demand-side cost sharing by requiring the patient to share in the cost of treatment, and/or (2) supply-side cost sharing which seeks to alter the incentives to health care providers to provide certain services (Ellis and McGuire, 1993). Capping the overall reimbursement per patient and quarter, like it is practice for self-employed ambulatory care physicians in Germany, may act as an example for supply-side cost sharing.

Given the imperfection of the health care market there might still be the discussion if consumer and producer surpluses can be applied. However, even if there is monopoly and uniform pricing, there remains a consumer surplus. In order to completely extract the consumer surplus there needs to be perfect price discrimination. Even if there are many suppliers offering different contracts (multi-part tariffs and different qualities), they will typically not be able to extract the entire consumer surplus in health care (Cabral, 2002). What about the producer surplus? If the supply (marginal cost) curve is more or less horizontal, there is no producer surplus. If competition is fierce one would expect the producer surplus being small. Similarly, if those purchasing health services are good negotiators (good at designing contracts), the contracts should be such that suppliers only earn a normal profit (i.e., don’t earn excess profits); they have to supply excellent quality without making extranormal profits. If suppliers earn a producer surplus there might be the possibility of underestimating (or providing a lower bound for) the social surplus (Cabral, 2002).

Different limitations directly apply to the simplistic examples: For the UK calculations a lot of assumptions have been taken into account for which some are quite strict: The cost of a HRG not clearly be defined: how can we compare the annual (daily) cost for one nurse with the revenue that hospital is getting through the HRGs, if we do not know how many patients are visiting the emergency room on average per day? Of course there might be more patients being administered during days than during nights, on the other hand there might also be more staff in the emergency unit during days than during nights. Anyhow, for simplicity and a theoretical calculation we assumed that the revenue from one HRG can be compared with the cost per nurse even though that one nurse might be able to treat more than one patient per day. This discussion strongly suggests further research in this area including a primary research component including potentially a micro costing approach.

Limitations of this research include the simplicity of the analyses and the secondary research nature of it. However, the purpose of this work was to analyze the existence of convenience in health care from a conceptual perspective based on a strong economic theory. To the best of our knowledge there are currently no similar research results available. Hopefully, the work published in this research paper will initiate further theoretical as well as empirical studies.

## Conclusion

The analyzed examples in this article support the underlying hypothesis for this research: “Value of convenience defined as the consumer surplus in health care can be shown in different health care settings.” Again, this hypothesis should be accepted as a starting point in this research area and hence further primary research is strongly recommended in order to fully proof this concept.

## Key Points for Decision Makers

- The analyzed examples in this article support the underlying hypothesis for this research: “Value of convenience defined as the consumer surplus in health care can be shown in different health care settings.”
- To the best of our knowledge there are currently no similar research results available.
- The work published in this research paper should initiate further theoretical as well as empirical studies.

## Conflict of Interest Statement

Dr. Walzer and Dr. Nuijten received a research fund for the underlying research. Drs. Wiener and Dr. Oertel are employed at F. Hoffmann-La Roche AG.
